# Prognosis and Tumour Immune Microenvironment of Patients With Hepatocellular Carcinoma by a Novel Pyroptosis-Related lncRNA Signature

**DOI:** 10.3389/fimmu.2022.836576

**Published:** 2022-06-24

**Authors:** Ze Zhang, Jin Shang, Bingyang Hu, Huizhong Shi, Yinbiao Cao, Junfeng Li, Tianyu Jiao, Wenwen Zhang, Shichun Lu

**Affiliations:** ^1^Medical School of Chinese People’s Liberation Army (PLA), Beijing, China; ^2^Faculty of Hepato-Pancreato-Biliary Surgery, Chinese PLA General Hospital, Beijing, China; ^3^Institute of Hepatobiliary Surgery of Chinese PLA, Beijing, China; ^4^Key Laboratory of Digital Hepatobiliary Surgery, PLA, Beijing, China

**Keywords:** hepatocellular carcinoma, pyroptosis, long non-coding RNAs, prognostic, tumour immune microenvironment, immune checkpoint inhibitors

## Abstract

Worldwide, hepatocellular carcinoma (HCC) is the most common subtype of liver cancer. However, the survival rate of patients with HCC continues to be poor. The recent literature has revealed that long non-coding RNAs (lncRNAs) and the occurrence of pyroptosis can perform a substantial task in predicting the prognosis of the respective condition along with the response to immunotherapy among HCC patients. Thus, screening and identifying lncRNAs corelated with pyroptosis in HCC patients are critical. In the current study, pyroptosis-related lncRNAs (PR-lncRNAs) have been obtained by co-expression analysis. The Least Absolute Shrinkage and Selection Operator (LASSO) and univariate and multivariate Cox regression assessments have been performed to develop a PR-lncRNA prognostic model. The risk model was analysed using Kaplan–Meier analysis, principal component analysis (PCA), functional enrichment annotation, and a nomogram. The risk model composed of five PR-lncRNAs was identified as an independent prognostic factor. The tumour immune microenvironment (TIME) was assessed using model groupings. Finally, we validated the five PR-lncRNAs *in vitro* using a quantitative real-time polymerase chain reaction (qRT-PCR).

## Introduction

The most common type of primary liver cancer is hepatocellular carcinoma (HCC) (1). The surgical option is not suitable for the majority of HCC patients with advanced stage (2). The efficacy of conventional systemic therapy for advanced HCC is limited (3). The development and progression of the condition get regulated by an individual’s immune system and can thus play a vital function in the preliminary analysis of the disease (4). Immune checkpoint inhibitors (ICIs) combined with anti-angiogenic agents have recently been shown to significantly prolong the survival time of patients with unresectable HCC (5, 6). The major unresolved challenge in HCC immunotherapy is identifying and validating predictive biomarkers (7).

Pyroptosis is a lytic type of regulated cell death correlated with inflammation induced by proinflammatory signalling molecules including ATP, IL-1β, IL-18, and high mobility group protein B1 (HMGB1) (8). This kind of cell death has been found prominent in inflammatory cells, such as macrophages, and can be triggered by bacterial or pathogenic infections. Several studies suggested a strong correlation between pyroptosis and the tumour immunological microenvironment (TIME). On the one hand, inflammasome-mediated pyroptosis activation and pyroptosis-produced cytokines alter the immune microenvironment by evading immune surveillance, thus promoting tumour development; conversely, pyroptotic cytokines can have a chemotactic effect on immune cells, keeping the tumour immune microenvironment activated and improving the efficacy of tumour immunotherapy (9). Gasdermin E (GSDME) is one of the important proteins that mediate cellular pyroptosis and has been found to act as a tumour suppressor by activating tumour pyroptosis, thereby enhancing the antitumour immune effect (10). Furthermore, recent studies have discovered that long non-coding RNAs (lncRNAs) can either induce or inhibit pyroptosis. Silencing the lncRNA HOTTIP, for example, could inhibit cell proliferation and NLRP1 inflammasome-mediated pyroptosis (11). In mouse macrophages, lncRNA Neat1 promotes the assembly of inflammasomes such as NLRP3, NLRC4, and AIM2 and the subsequent processing of pre-caspase-1 (12). By stabilizing mature caspase-1, Neat1 also promotes pyroptosis and the production of IL-1β (12). Nevertheless, a very limited number of studies have been conducted on the PR-lncRNAs in HCC. Likewise, the prognostic significance of PR-lncRNAs and their correlation with TIME in HCC is still yet not fully defined.

The current study entails the identification and validation of a prognostic model among HCC patients based on PR-lncRNAs. Following that, we developed and evaluated a nomogram to predict the OS of patients with HCC. Then, we explored the differences in the TIME between high-risk and low-risk groups, which provides a functional value for immunotherapy of HCC. Finally, we confirmed that the expression of the five PR-lncRNAs in the model differed significantly between HCC and normal tissues using quantitative real-time polymerase chain reaction (qRT-PCR).

## Materials and Methods

### Acquisition of Data in Patients With HCC

The Cancer Genome Atlas (TCGA) database was employed for obtaining the transcriptomics and clinical data with a sample size of 377 patients affected with HCC. The lncRNA matrix was annotated and extracted from RNA-seq datasets using the “Perl” language. The exclusion criteria of patients in the given study were measured over the survival time of patients less than those of 30 days or those who have missing expression data (n = 35).

### Identification Pyroptosis-Related Genes and PR-lncRNAs

The UCSC’s Xena program was used to obtain the copy number variation (CNV) data which are also publicly available under certain guidelines. The MSigDB Team (REACTOME PYROPTOSIS) (http://www.broad.mit.edu/gsea/msigdb/) and previous studies (13) were employed to retrieve 52 pyroptosis-related genes (PRGs) (**Supplementary Table 1**). The “RCircos” R program was used to map the environment of genomic CNVs in chromosomes. The corelation between PRGs and the lncRNAs was identified using Pearson’s correlation analysis while keeping |Pearson R| > 0.4 and P < 0.001. We conducted a differential expression analysis of PR-lncRNAs using the P < 0.05 and |log2FC| ≥ 1 criterion.

### Construction and Validation of the Risk Signature

The screening process was conducted using the univariate Cox regression (P < 0.05) analysis to further investigate the correlation of PR-lncRNAs to OS among HCC patients. The complete dataset enrolled for training and testing at a ratio of 7:3 was chosen randomly employing the R project “caret” package. Likewise, no statistically significant variations in clinical characteristics between the two datasets were recorded (P > 0.05).

A PR-lncRNA model was established using a training set and validated by comparing the established signatures present among the entire dataset and the testing dataset. LASSO regression analysis was conducted for the training group, employing the R project “glmnet” package to further select the screened lncRNAs (employing the penalty parameter determined through 10-fold cross-validation), and the optimal lambda was obtained when the partial likelihood deviance reached the minimum value. Finally, the PR-lncRNA signature was determined using multivariate Cox regression analysis and the lowest Akaike information criterion (AIC) value (14). The formula Risk score = coef (lncRNA1) * expr (lncRNA1) + coef(lncRNA2) * expr (lncRNA2) +… + coef (lncRNAn) * expr(lncRNAn) was used to calculate the risk score for each HCC patient. In addition, the lower and higher risk categories were defined over the median risk score. The R package “survival” was used to perform Kaplan–Meier survival analysis to compare overall survival (OS) between high-risk and low-risk groups. The signature’s predictive capabilities were then assessed by using calibration and time-dependent receiver operating curves (ROC) obtained through the experiment. In correspondence, the concordance index (C-index) was estimated to assess the model’s discrimination. The principal component analysis (PCA) effectively reduces dimensionality, identifies models, and visualizes groupings of high-dimensional data of entire gene expression profiles, 52 PRGs, PR-lncRNAs, and the risk model based on the expression patterns of the five PR-lncRNAs.

### Nomogram Construction and Assessment

Based on the five-PR-lncRNA signature and HCC clinical staging, a nomogram was developed using “rms,” a package of R. Later, ROC, calibration curves, and the C-index were created to evaluate the predictive capability of the nomogram. Likewise, the study has also made the assessment based on the clinical benefits of the nomogram by performing decision curve analysis (DCA) along with other clinical features.

### The Tumour Immune Microenvironment Analysis

Similarly, the Cell-type Identification By Estimating Relative Subsets Of RNA Transcripts (CIBERSORT) technique was applied for the quantitative assessment of the tumour tissue transcriptomic data and later translated into an absolute abundance of immune and stromal cells (15, 16). This study examined 22 different types of human immune cells. Furthermore, a single-sample GSEA (ssGSEA) approach was introduced employing gene set variation analysis (GSVA) and the “GSEABase” package to evaluate the extent of pyroptosis, inflammatory infiltration profiles, and immune functions (17). In addition, Wilcoxon analysis was used to compare the level of immune cells infiltrating among the high-risk and low-risk groups. Finally, we assessed the expression of 47 immune checkpoint genes in both groups.

### Functional Enrichment Analysis and Sensitivity to Immunotherapy in the High- and Low-Risk Groups

The functional assessment was performed to understand the signal transduction pathways by using gene set enrichment analysis (GSEA computer program, version 4.1.0) (18). The current analysis comprises gene sets (i.e., c2.cp.kegg.v7.4.symbols.gmt (19) and h.all.v7.4.symbols.gmt (18)) while keeping the screening criterion to a nominal P-value < 0.05. We then used the Gene Ontology (GO) analysis to identify functional differences in differentially expressed genes between the high-risk and low-risk groups (20). This procedure utilized the R package clusterProfiler (21). The analysis threshold was determined by P-values, with P<0.05 indicating significantly enriched functional annotation. Subsequently, we used the Tumour Immune Dysfunction and Rejection (TIDE) algorithm to predict the difference in sensitivity to immunotherapy between the high- and low-risk groups (22).

### Isolation of RNA and qRT-PCR Analysis

Total RNA was extracted from 20 pathologically confirmed tumour tissues and normal liver tissues from HCC patients who had not received preoperative antitumour therapy. The specimen obtained immediately after excision from the respective patients were rapidly frozen in liquid nitrogen (-196°C). The procedure of RNA extraction involves the application of complementary DNA (cDNA) which was constructed by using the PrimeScript Reverse Transcriptase Reagent Kit (Takara Bio, Inc., Japan) and TRIzol Reagent (Invitrogen, CA, USA). Similarly, the detection and amplification of the respective genes were conducted using TB Green^®^ Premix Ex Taq™ II (Takara, Tokyo, Japan) in the ABI Step One Plus Real-Time PCR system (Applied Biosystems). At the same time, β-actin was kept as an endogenous control. Furthermore, normalization of the expression levels (i.e., MKLN1-AS, HPN-AS1, MED8-AS1, ZNF232-AS1, and SREBF2-AS1 in accordance with β-actin) was conducted by using the 2^−ΔΔCt^ approach. The primer sequences are given in **Supplementary Table 2**. The conducted study was confirmed through the board of ethical committee of the Chinese PLA General Hospital (approval no. S2018-111-01). A written consent form was taken from all patients.

### Statistical Analysis

The data obtained from the aforementioned procedures were analysed using R version 4.1.0 (Institute of Statistics and Mathematics, Vienna, Austria), “Perl” language, and GraphPad Prism 8 (GraphPad Software Inc, La Jolla, CA, USA). Survival analysis was performed using K-M and log-rank tests. The t-test or Mann–Whitney U test was used to compare two independent groups. The χ^2^ test was employed to analyse categorical data. P < 0.05 was regarded as statistically significant (*P < 0.05, **P < 0.01, ***P < 0.001).

## Results

### Expression and Copy Number Variations of Pyroptosis Genes in HCC

**Figure 1:**
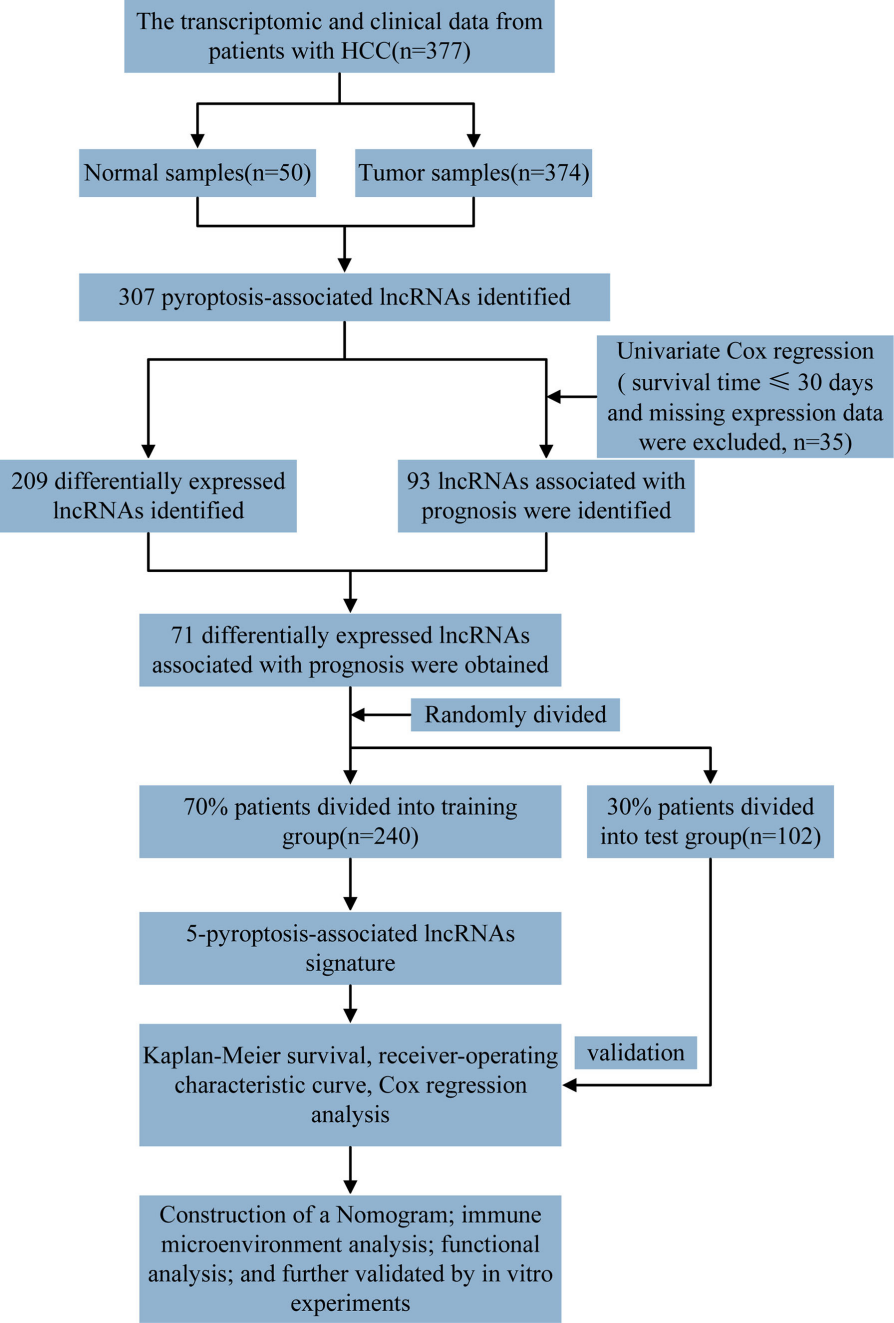
Representation of the overall flowchart of the given study.

**Figure 2:**
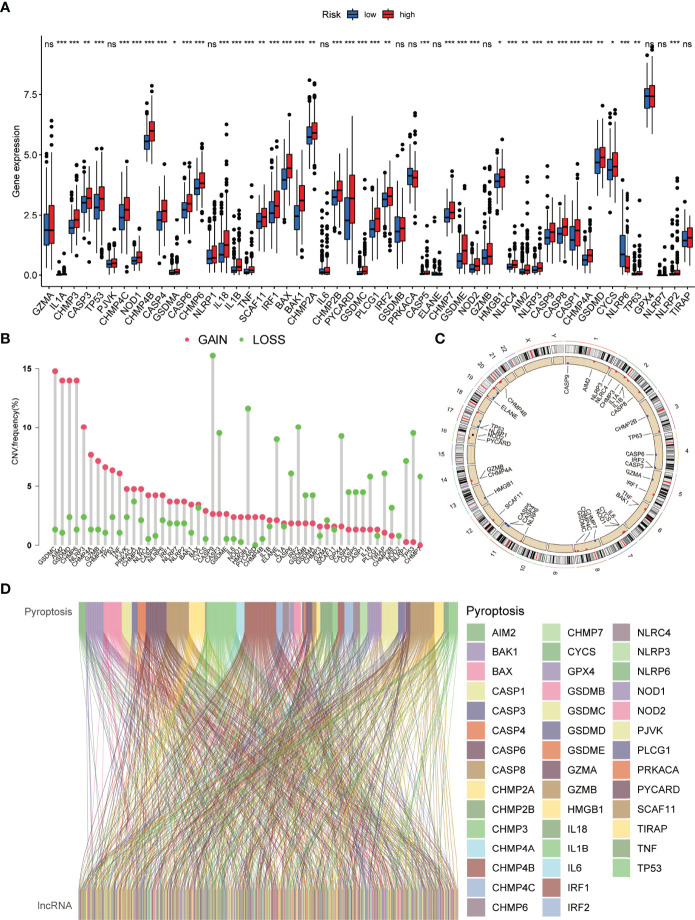
Genetic and expression variation of the PRG gene in HCC. **(A)** Comparative analysis of normal and HCC tissues based on 52 PRGs. **(B)** The CNV frequency of 52 PRGs in the HCC cohort. **(C)** Assessment of the 23 chromosomes to identify the location of CNVs among 52 PRGs in the HCC cohort. **(D)** Sankey diagram depicting the relationships between 52 PRGs and PR-lncRNAs (ns, no significance; *P < 0.05; **P < 0.01; ***P < 0.001).

The detailed workflow diagram for risk prediction model construction and corresponding analyses is presented in **Figure 1**. The study included 52 PRGs of which the mRNA expression was compared between the normal and HCC tissues. The results concluded a relatively higher expression of PRGs in tumour tissues than normal ones (**Figure 2A**). In addition, somatic copy variations among the respective PRGs were studied. A significant number of copy number alterations were discovered among all PRGs including CHMP6, GSDMC, AIM2, NLRP3, and GSDMD which have had enhanced extensive CNVs. At the same time, TP53, CASP9, ELANE, CASP3, GPX4, HMGB1, and IRF2 presented an overall decrease in the CNV (**Figure 2B**). The corresponding locations of every CNV on the chromosomes are shown in **Figure 2C**. The entire TCGA database was used to extract the matrix expression of 52 PRGs and 4,668 lncRNAs. **Figure 2D** depicts the co-expression network of PRGs and PR-lncRNAs using a Sankey diagram. Eventually, 307 PR-lncRNAs were identified (| Pearson R | > 0.4, P < 0.001).

### Development of a Risk Model Based on PR-lncRNAs Along With Validation Among HCC Patients

**Figure 3:**
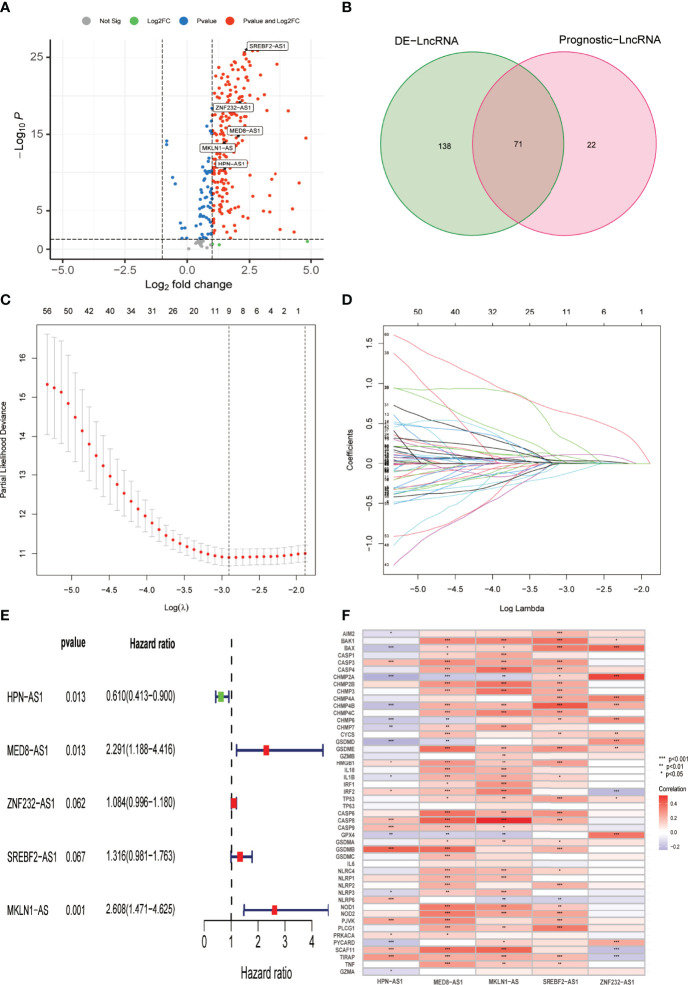
Establishment of the PR-lncRNA prognostic signature for HCC patients. **(A)** Volcano plot of differentially expressed PR-lncRNAs in HCC from entire set. **(B)** Venn diagram to identify differentially expressed PR-lncRNAs associated with prognosis. **(C, D)** Ten-fold cross-validation by the LASSO Cox regression for the prognostic value of the PR-lncRNAs in the training set. **(E)** The presentation of five lncRNAs in multivariate Cox regression analysis. **(F)** Representation of the correlations between 52 PRGs and the five prognostic PR-lncRNAs *via* heatmap.

According to the conventional P < 0.05 and |log2FC| > 1 criteria, 209 PR-lncRNAs were identified as differentially expressed from 307 PR-lncRNAs (**Figure 3A**). Then, using univariate Cox regression analysis, 71 pyroptosis-associated prognostic lncRNAs were filtered from the entire dataset (**Figure 3B**, **Supplementary Table 3**). Following that, in 7:3 ratios, there was a random distribution of 342 HCC patients to the training and testing groups with similar clinical characteristics in all three groups (**Table 1**). The optimal model in the training dataset was identified using LASSO regression and Cox proportional hazard model analysis (**Figures 3C, D**). In addition, multivariate analysis identified five PR-lncRNA risk scores (**Supplementary Table 4**), which were calculated using the following formula:

**Table 1:** Clinical features of 342 patients with hepatocellular carcinoma.

Character	Training dataset	Testing dataset	Entire dataset	P-value
	n = 240	n = 102	n = 342	
**Age**				0.928
≤65	150	66	216	
>65	90	36	126	
**Gender**				0.819
Female	74	35	109	
Male	166	67	233	
**Grade**				0.652
G1–G2	147	67	214	
G3–G4	88	35	123	
Unknown	5	0	5	
**TNM stage**				0.887
I–II	166	72	238	
III–IV	61	22	83	
Unknown	13	8	21	
**Tumour stage**				0.730
T1–T2	173	79	252	
T3–T4	64	23	87	
Unknown	3	0	3	

*Riskscore* = -0.49 * expr HPN-AS1 + 0.83 * expr MED8-AS1 + 0.96 * expr MKLN1-AS + 0.27 * expr SREBF2-AS1 + 0.08 * expr ZNF232-AS1). **Figure 3E** describes the correlation between each lncRNA and OS, while **Figure 3F** illustrates the relationship between five PR-lncRNAs and PRGs across the entire TCGA dataset.

**Figure 4:**
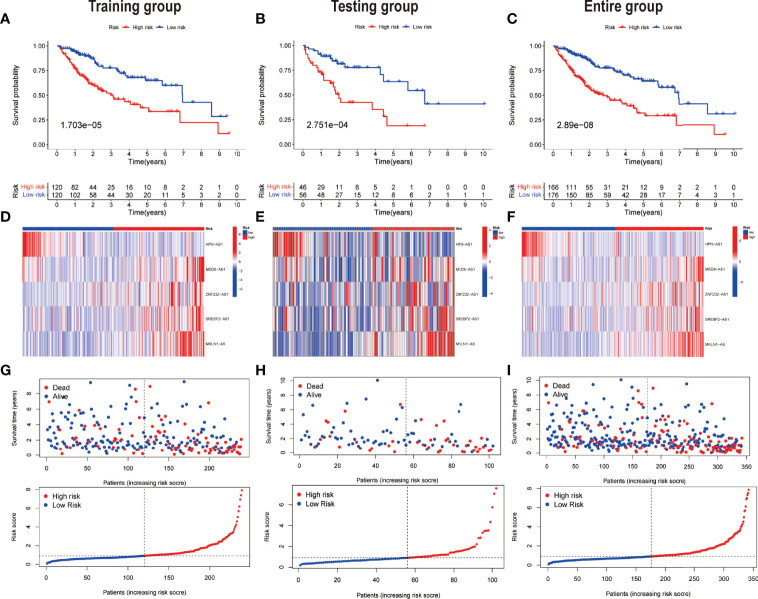
Prognostic value of the risk model of the five PR-lncRNAs. The assessment of overall survival rate among low-risk and high-risk groups of HCC by employing Kaplan–Meier survival curves for the **(A)** training, **(B)** testing, and **(C)** entire set. The presentation of the level of expression of five prognostic lncRNAs *via* clustering analysis heatmap of each patient enrolled in **(D)** training, **(E)** testing, and **(F)** entire set. The coherence of survival time and survival status among the low-risk and high-risk groups and the distribution of PR-lncRNA model-based risk score for the **(G)** training, **(H)** testing, and **(I)** entire set.

Taking into account the median of the risk scores, HCC samples were classified into low-risk and high-risk groups in the training dataset. Subsequently, for each patient, the uniform formula was applied to calculate risk ratings in the test and the complete dataset. The survival study revealed that the OS among the high-risk groups was relatively shorter than that of the low-risk groups (P = 1.703e -05) (**Figure 4A**). Likewise, in the testing dataset (**Figures 4B**) and the entire dataset (**Figures 4C**), cases in the low-risk group had more remarkable overall survival compared to those of the high-risk group as per the survival analysis. **Figures 4D–F** show the relative expression of the five PR-lncRNAs for each patient in each of the three groups, whereas **Figures 4G–I** represent the status of survival and risk score distribution of risk scores of HCC cases in the low-risk and high-risk groups.

### Prognostic Risk Model Evaluation

**Figure 5:**
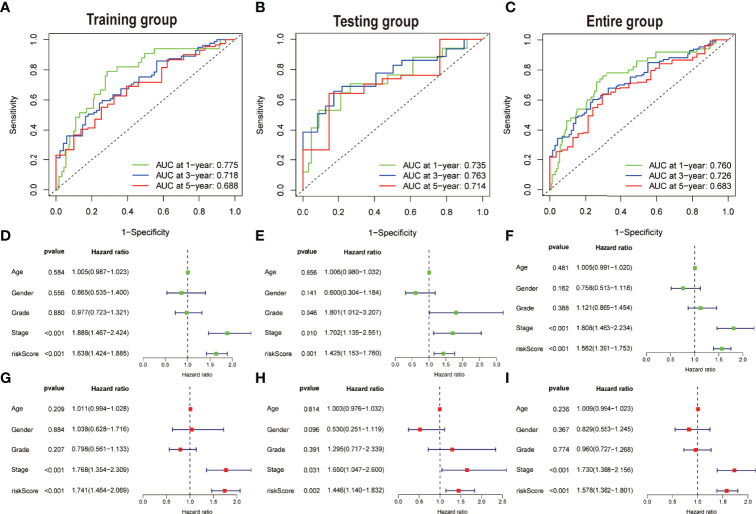
Assessing the accuracy and independence of the model. Curves of receiver operating characteristic (ROC) related to prognostic signature for predicting the 1, 3, and 5 years of survival in the **(A)** training, **(B)** testing, and **(C)** entire set. Univariate Cox regression results in the **(D)** training, **(E)** testing, and **(F)** entire set. Multivariate Cox regression results in the **(G)** training, **(H)** testing, and **(I)** entire set.

The area under the curve (AUC) was evaluated in each of the three groups. The AUC values of the 1, 3, and 5 years of OS were 0.775, 0.718, and 0.688, respectively, in accordance with the training set (**Figure 5A**). The AUC values of the OS were found out to be 0.735, 0.763, and 0.714 for the 1, 3, and 5 years of survival in the test dataset, respectively (**Figure 5B**), whereas the AUC values for OS in the total dataset were calculated as 0.760, 0.726, and 0.683 for the 1, 3, and 5 years of survival, respectively (**Figure 5C**). In addition, univariate and multivariate Cox regression assessments were performed to investigate whether the risk model had independent predictive characteristics for HCC. According to univariate Cox regression assessment, the hazard ratio (HR) of the model and the 95% confidence interval (CI) were found out to be 1.638 and 1.424–1.885 (P < 0.001), respectively (**Figure 5D**), in the training set. The HR in the univariate Cox regression assessment outcomes for the testing and complete datasets were reported as 1.425 (95% CI: 1.153–1.760, P = 0.001) and 1.562 (95% CI: 1.391–1.753, P < 0.001), accordingly (**Figures 5E, F**). In the training set, the analysis of multivariate Cox regression has divulged that the HR was found out to be 1.741, with a 95% CI of 1.464–2.069 (P < 0.001) (**Figure 5G**). The HR in the analysis of multivariate Cox regression outcomes for the testing and complete datasets was calculated as 1.446 (95% CI: 1.140–1.832, P = 0.002) and 1.578 (95% CI: 1.382–1.801, P < 0.001), accordingly (**Figures 5H–I**). These findings imply that clinicopathological variables including TNM stage, age, and gender have an insignificant impact on the risk model.

### Comparing the Accuracy and Discrimination of Prognostic Risk Model With Clinical Characteristics

**Figure 6:**
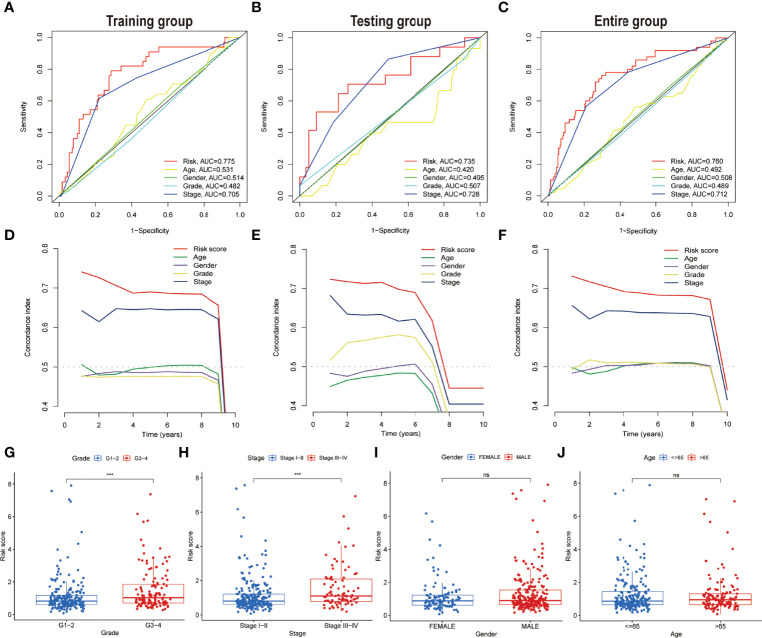
Comparing the prognostic precision of the model and clinical features and comparing risk scores between subgroups with different clinical characteristics. **(A–C)** Prognostic accuracy of the risk scores, age, gender, grade, and tumour stage were compared using time-dependent ROC curves in the time duration of 1 year. **(D–F)** The concordance index was used for comparing the discrimination of the model, age, gender, grade, and tumour stage in three datasets. Risk scores are grouped by different clinical characteristics in the entire set. **(G)** Grade. **(H)** Stage. **(I)** Gender. **(J)** Age. ns, no significance; ***P < 0.001.

To assess the credibility of the model, the factors such as tumour stage, age, gender, and pathological grade were enlisted as candidate predictive variables to monitor whether the risk score model could be a suitable predictor of survival. Further, the AUC curves for 1-year prognosis and the C-index were evaluated in the three datasets. Among these characteristics, it was discovered that the model had the highest AUC values (**Figures 6A–C**). Similarly, among other clinical markers, the C-index of the risk score was found to be relatively larger with time (**Figures 6D–F**), implying more reliable predictability of the model in assessing the extent of the HCC prognosis. Additionally, the risk scores were considerably more significant in tumour grades 3–4 (P < 0.001) or tumour stages III–IV (P < 0.001) than in tumour grades 1–2 or tumour stages I–II upon monitoring the link between risk scores and clinical characteristics of HCC patients (**Figures 6G, H**). However, not a significant association was observed between risk score and the parameters such as gender or age (**Figures 6I, J**).

### Principal Component Analysis Further Confirms the Grouping Ability of the Signature

**Figure 7:**
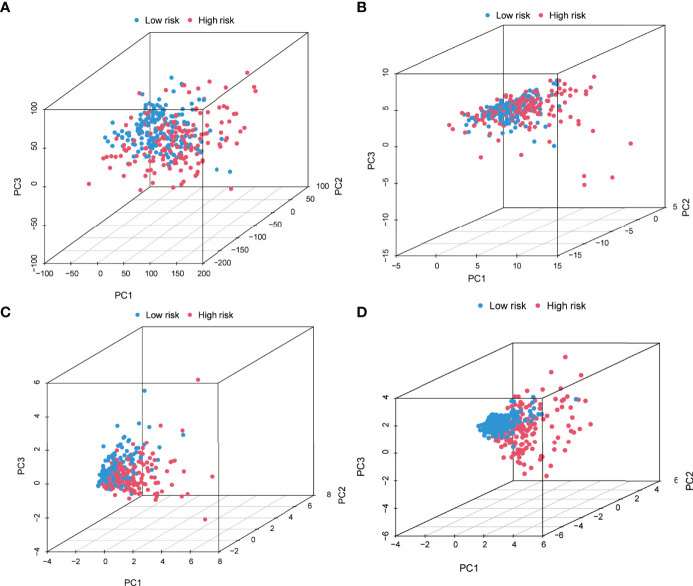
Principal component analysis between the high-risk and low-risk groups based on **(A)** entire gene expression profiles, **(B)** 52 PRGs, **(C)** PR-lncRNAs, and **(D)** risk model based on the five PR-lncRNAs in the entire set.

PCA was executed based on the entire genome expression profile of 52 PRGs and five PR-lncRNAs, and the risk model based on the expression of five PR-lncRNAs to identify differences among the low-risk and high-risk groups. The relative dispersion of the distribution based on the entire genome expression profile of 52 PRGs and five PR-lncRNAs among the high-risk and low-risk groups is presented in **Figures 7A–C**, respectively. However, the outcomes of the current model imply that the distribution of low-risk and high-risk groups is distinct (**Figure 7D**). These findings elucidate that the prognostic model could distinctly identify between the two groups.

### Development and Assessment of the Nomogram

**Figure 8:**
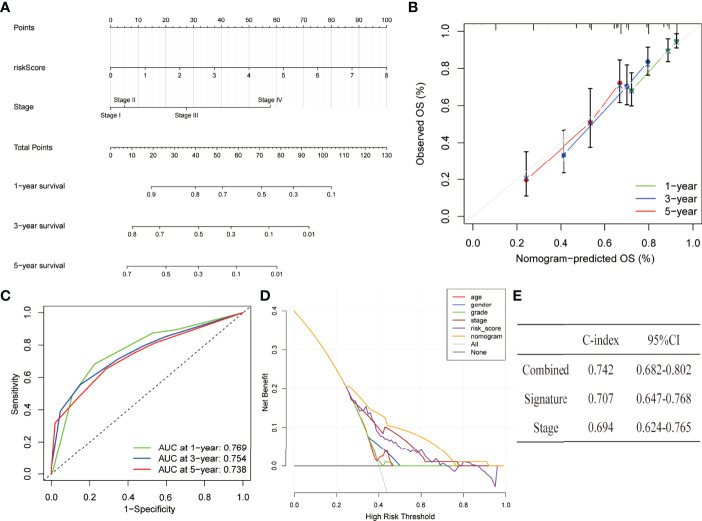
Establishment and assessment of a nomogram in the entire set. **(A)** The nomogram predicts the probability of the 1, 3, and 5 years of OS. **(B)** The calibration plot prediction *via* nomogram of the OS at 1, 3, and 5 years. **(C)** ROC curves of the nomogram for predicting the 1, 3, and 5 years of survival. **(D)** Decision curve analysis for the nomogram, age, gender, grade, stage, and risk score. **(E)** C-index of the nomogram, signature, and stage.

The nomogram has integrated the risk model and tumour stage information in the entire dataset to predict the 1, 3, and 5 years of overall survival (**Figure 8A**). The calibration curves of the 1, 3, and 5 years of survival probability have revealed a good consistency between nomogram predictions and actual observations (**Figure 8B**). The AUC values obtained for the 1, 3, and 5 years of overall survival were 0.769, 0.754, and 0.738, respectively (**Figure 8C**). The constructed nomogram also has a higher prediction accuracy than a risk model alone. Furthermore, to assess the clinical application of the nomogram, DCA was performed. The results have concluded that the nomogram was able to outperform both the risk model and clinical characteristics in terms of net benefit (**Figure 8D**). Similarly, the C-index parameters were applied to evaluate the nomogram’s discriminative strength which showed a value of 0.742 which is higher in comparison to the C-index of the stage (0.694) and signature (0.707) alone (**Figure 8E**).

### Evaluation of TIME and Checkpoint Genes Between the High-Risk and Low-Risk Groups

**Figure 9:**
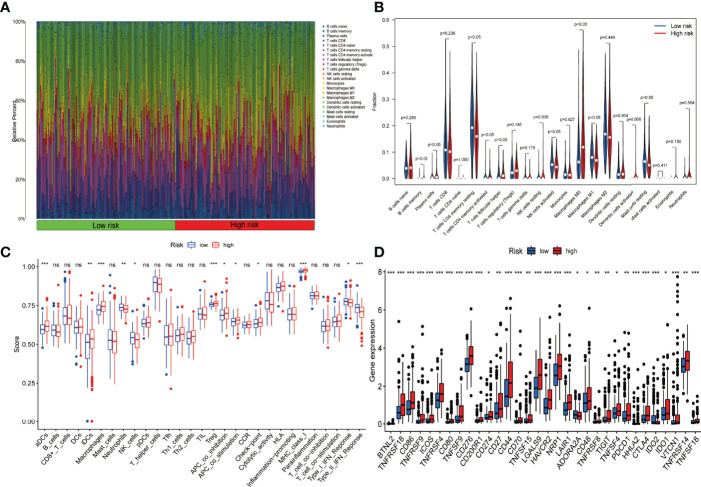
Presentation of the expression profile of immune checkpoints genes and Immune cell infiltration among low-risk and high-risk groups. **(A)** Barplot depicting the 22 immune infiltrating cell proportions of the high-risk and low-risk. **(B)** The differential proportion of the immune cells has been calculated *via* violin plot among low-risk and high-risk patients. **(C)** Boxplots depict the 29-immune signature ssGSEA scores of high-risk patients compared to low-risk patients. **(D)** Comparative analysis among the high- and low-risk categories on the basis of the expression profile of immune checkpoint genes (ns, no significance; *P < 0.05; **P < 0.01; ***P < 0.001).

Each fraction type of tumour-infiltrating immune cell was calculated by applying the CIBERSORT method in all HCC patients. The proportions of distinct tumour-infiltrating immune cells differed significantly in both groups (**Figure 9A**). **Figure 9B** illustrates a much lower proportion of resting mast cells, M1 macrophages, and tumour-infiltrating activated NK cells among high-risk patients. On the contrary, the fraction of M0 macrophages and tumour-infiltrating follicular helper T cells was much more significant in high-risk cases. The ssGSEA approach was also utilized to analyse the tumour immune microenvironment. Immune cells including aDCs, iDCs, macrophages, neutrophils, NK cells, and Tregs were discovered to differ significantly between the two groups (**Figure 9C**). In addition, low-risk patients showed more cytolytic activity, as measured by type II IFN and type I IFN responses. In contrast, high-risk patients had the opposite effect for APC co-inhibition, checkpoint activation, and MHC class I expression (**Figure 9C**). Considering the relevance of ICIs in treating HCC, the differential expression analysis of genes involved in immune checkpoints was conducted among the high-risk and low-risk groups. The results concluded that cases in the high-risk group had an elevated expression of these genes (i.e., CD-274 (PD-L1), PDCD-1 (PD-1), and CTLA4) in comparison to the low-risk category (**Figure 9D**).

### Functional Enrichment Analysis, Sensitivity to Immunotherapy Between High- and Low-Risk Groups, and Verification of the Prognostic LncRNA Expression by qRT-PCR

**Figure 10:**
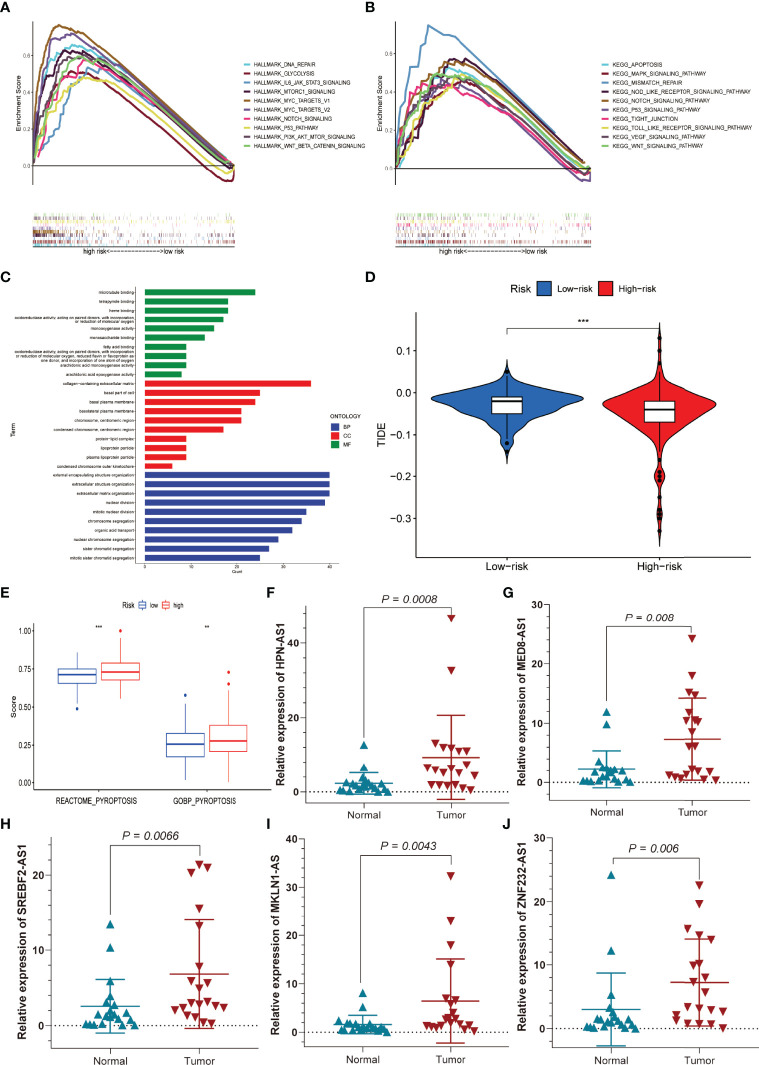
Functional enrichment analysis, sensitivity to immunotherapy, and pyroptosis scores for the high- and low-risk groups, and external experimental validation of five PR-lncRNAs. **(A)** Hallmark gene set. **(B)** KEGG, Kyoto Encyclopedia of Genes and Genomes. **(C)** GO analysis. **(D)** TIDE score for the high- and low-risk groups. **(E)** Differences in pyroptosis scores between the low and high-risk groups. **(F–J)** The experiment confirmed the difference in the prognostic lncRNA expression between HCC and adjacent non-tumour tissues. **P < 0.01; ***P < 0.001.

The hallmark gene sets and KEGG pathway enrichment analysis were conducted using GSEA between the high-risk and low-risk groups. P53, NOTCH, PI3K-AKT-MTOR, MTORC1, DNA repair, glycolysis, IL-6-JAK-STAT3, MYC-Targets-V1, MYC-Target-V2, and WNT-β-Catenin pathways were significantly enriched in the high-risk group, according to GSEA of the hallmark gene sets (**Figure 10A**). Likewise, MAPK, NOD-like receptor, P53, TIGIT junction, VEGF, apoptosis, mismatch repair, Toll-like receptor, and WNT-signalling pathways were significantly enriched in KEGG pathway enrichment analysis among high-risk patients (**Figure 10B**). As shown in **Figure 10C**, interestingly, GO enrichment analysis showed that differentially expressed genes between the low- and high-risk groups were mainly enriched in lipid metabolism and cell proliferation, such as fatty acid binding, arachidonic acid epoxygenase activity, lipoprotein particle, protein–lipid complex, nuclear division, and mitotic nuclear division. We subsequently used TIDE to evaluate the efficacy of immunotherapy in both groups. The higher TIDE scores were associated with a higher likelihood of immune escape, indicating that patients were less likely to benefit from ICI therapy. Our results showed that the TIDE scores were lower in the high-risk group than in the low-risk group, meaning that the high-risk group would benefit more from immunotherapy than the low-risk group (**Figure 10D**). In addition, the “GSVA” package was employed to determine the extent of pyroptosis among both groups. We discovered that patients with high-risk HCC had higher pyroptosis scores (**Figure 10E**). Finally, we used qRT-PCR to confirm the distinct expression patterns of the five prognostic PR-lncRNAs in HCC and adjacent normal tissues. The results revealed that the expressions of HPN-AS1 (**Figure 10F**), MED8-AS1 (**Figure 10G**), SREBF2-AS1 (**Figure 10H**), MKLN1-AS (**Figure 10I**), and ZNF232-AS1 (**Figure 10J**) were relatively higher among HCC tissues than those of the normal tissues.

## Discussion

Immunotherapy has been known to play a significant role as a method to eradicate tumour cells based on immune checkpoint inhibitors among a subset of HCC patients (23). However, immunotherapy is still ineffective for a large number of patients. As a result, additional TIME research and the development of new predictive models are required. Pyroptosis-induced inflammation has been shown to stimulate antitumour immunity and synergistically with checkpoint inhibitors (24). Previous research has concentrated on developing prognostic signatures or immune microenvironments involving pyroptosis genes (25, 26). There have been fewer reports of lncRNAs linked to pyroptosis in HCC.

The current study has successfully developed and validated a novel signature consisting of five PR-related lncRNAs (i.e., SREBF2-AS1 and HPN-AS1, both of which are engaged in the development of prognostic models for ferroptosis and tumour mutation burden among patients with HCC) (27, 28). Similarly, MKLN1-AS, another signature marker, has been known to stimulate hepatocellular carcinoma cell proliferation, invasion, and migration through YAP1 (29). MKLN1-AS gene knockdown inhibited the proliferation, migration, and invasion of HCC *in vitro* and induced apoptosis (30). In addition, other lncRNAs of the model were identified for the first time. Hence, considering the intermediate-risk score, the cases affected with HCC have been categorized into low-risk and high-risk groups whereas the high-risk group presented poor clinical outcomes. The PR-related lncRNA signature was found to be an independent risk factor for OS in multivariate Cox regression analysis. The model so far has outperformed traditional clinical characteristics in terms of HCC survival prediction as per ROC analysis and C-index values. In addition, a nomogram was applied for assessment of the OS in the duration of the 1, 3, and 5 years of the HCC patients and observed a complete consistency among the anticipated and the obtained values. Furthermore, the established nomogram was found to be more accurate and reliable in the prognosis of HCC patients, as evidenced by the ROC curve, C-index, and DCA analyses. The risk model, which was based on five PR-lncRNAs that were independently related to OS, was reasonably accurate, and this prediction model could discover novel biomarkers for future research.

We investigated the variations in TIME and the expression of immune checkpoint genes between the high-risk and low-risk groups using the standard five-PR-lncRNA signature. The proportion of distinct tumour-infiltrating immune cells was calculated using the CIBERSORT and ssGSEA algorithms. The results revealed that, compared to the low-risk group, the infiltrated tumour-killing immune cells in the high-risk group’s HCC tissues, such as neutrophils, activated NK cells, and M1 macrophages, were significantly reduced. In contrast, a significant increase in the population of immune cells that account for the promotion of tumour proliferation and metastasis, such as aDCs, M0 macrophages, and Tregs, were observed (3, 31). Similarly, the high-risk group had an elevated checkpoint and MHC-class-I immunosuppressive activities following the ssGSEA data (32). Furthermore, immune checkpoint-associated genes were more significantly expressed in patients of the high-risk group compared to the low-risk group, which may provide valuable information for identifying patients with HCC who may respond to ICI therapy.

Correspondingly, immune-related signalling pathways, such as MTORC1, MYC-TARGETS-V1, MAPK, MYC-TARGETS-V2, NOTCH, P53, PI3K-AKT-MTOR, mismatch repair, TIGHT junction, VEGF, and WNT-β-Catenin, were found considerably enriched in the high-risk group as per GSEA results (33, 34). In parallel, the assessment of inflammation-related or pyroptosis pathways (i.e., Toll-like receptor, apoptosis, IL-6-JAK-STAT3, and NOD-like receptor) revealed a significant inclination among the high-risk group (35). GO analysis showed that the differential gene functions in the high- and low-risk groups were mainly enriched in lipid metabolism and cell proliferation, which was closely associated with pyroptosis (36, 37). Additionally, HCC patients in the high-risk group were more sensitive to immune checkpoint inhibitors therapy. The ssGSEA algorithm was also utilized to generate the pyroptosis scores for the low-risk and high-risk groups and discovered a total increase in pyroptosis among the high-risk group. The application of qRT-PCR further validated the former assessment to evaluate the expression profile of HPN-AS1, MED8-AS1, ZNF232-AS1, SREBF2-AS1, and MKLN1-AS genes in HCC tissues which were found out to be considerably higher than in normal tissues. Based on the findings, we believe that this prediction signature could give reliable immunological biomarkers for HCC immunotherapy.

Furthermore, our research adds to our understanding of the molecular biology of PR-related lncRNAs in HCC. We also recognize that this research has some weaknesses and limitations. External validation using other clinical datasets would be advantageous. As a result, we will collect more clinical samples and increase the sample size. In the future, we will try to validate the correctness of this signature by doing more external experiments to investigate the involvement of lncRNAs and their interactions with PRGs.

In conclusion, the current study provides a way to estimate the prognosis of HCC cases and may contribute to assessing the processes and mechanisms of pyroptosis-regulated lncRNAs. The recent work has also assessed the TIME of HCC patients, which might have a vital role in immunotherapy for HCC.

## Data Availability Statement

The original contributions presented in the study are included in the article/**Supplementary Material**. Further inquiries can be directed to the corresponding author.

## Ethics Statement

The studies involving human participants were reviewed and approved by the Ethics Committee of the Chinese PLA General Hospital (Approval No. S2018-111-01). The patients/participants provided their written informed consent to participate in this study.

## Author Contributions

ZZ, JS, and BH designed the study. ZZ and JS collected and analysed the data. ZZ was the major contributor in writing the manuscript. JS and BH provided technical support. BH, YC, TJ, JL, and HS were involved in surgery, patient care, specimen collection, and literature search. WZ and SL provided guidance and advice for this article. SL, who provided the financial and ideas support and critically reviewed the manuscript, was our corresponding author. ZZ was responsible for writing the manuscript. SL was responsible for the analysis of data, data interpretation, and revision. All authors contributed to the article and approved the submitted version.

## Conflict of Interest

The authors declare that the research was conducted in the absence of any commercial or financial relationships that could be construed as a potential conflict of interest.

## Publisher’s Note

All claims expressed in this article are solely those of the authors and do not necessarily represent those of their affiliated organizations, or those of the publisher, the editors and the reviewers. Any product that may be evaluated in this article, or claim that may be made by its manufacturer, is not guaranteed or endorsed by the publisher.

## Abbreviations

HCC, hepatocellular carcinoma; TCGA, The Cancer Genome Atlas; PRGs, pyroptosis-related genes; PR-lncRNAs, pyroptosis-related long non-coding RNAs; LASSO, Least Absolute Shrinkage and Selection Operator; OS, overall survival; CI, confidence interval; PCA, principal component analysis; GSDMC, gasdermin C; GSDMD, gasdermin D; GSDME, gasdermin E; TIME, tumour immune microenvironment; DCA, decision curve analysis; CNVs, copy number variations; ICIs, immune checkpoint inhibitors; ssGSEA, single-sample GSEA; GSVA, gene set variation analysis; CIBERSORT, Cell-type Identification By Estimating Relative Subsets Of RNA Transcripts; qRT-PCR, quantitative real-time polymerase chain reaction; cDNA, complementary DNA; ROC, receiver operating characteristic; AUC, area under curve; AIC, Akaike information criterion.
