# Supplementary material for: Prognosis and Tumour Immune Microenvironment of Patients With Hepatocellular Carcinoma by a Novel Pyroptosis-Related lncRNA Signature
Source: Front Immunol. 2022 Jun 24;13:836576. doi: 10.3389/fimmu.2022.836576 (PMC9263208; doi:10.3389/fimmu.2022.836576)
Supplement: Supplementary file 2 [file Table_2.docx]

**Table S2. The primer sequences of the five PR-lncRNAs.**

**HPN-AS1** forward: 5’- TCAGGTCGGACCAATCAACAG -3’

reverse: 5’- GAGCAGCCAACTTAGGACTTC -3’;

**MED8-AS1** forward: 5’- GCATGGAAGCCGACTTGATGTTG -3’

reverse: 5’- ACTATTCTCCCATGTCACTTCTT -3’;

**MKLN1-AS** forward: 5’- GTGCTGCACCAGACATTATAACC -3’

reverse: 5’- AGGCAGTTCCTGAGTTATTTACC -3’;

**SREBF2-AS1** forward: 5’- CTTGCGCAGGTGGAGAGTGA -3’

reverse:5’- CCTTGCAGGCATGTATGATGG -3’;

**ZNF232-AS1** forward: 5’- ATCTGCACAACGGGGATGTA -3’

reverse:5’- GCTCAGGTCTAATGCAGCACC -3’;

**β-actin** forward: 5’-TGACGTGGACATCCGCAAAG-3’

reverse: 5’-CTGGAAGGTGGACAGCGAGG-3’.
