# Supplementary material for: Prognosis and Tumour Immune Microenvironment of Patients With Hepatocellular Carcinoma by a Novel Pyroptosis-Related lncRNA Signature
Source: Front Immunol. 2022 Jun 24;13:836576. doi: 10.3389/fimmu.2022.836576 (PMC9263208; doi:10.3389/fimmu.2022.836576)
Supplement: Supplementary file 3 [file Table_3.docx]

| gene | HR | HR.95L | | HR.95H | | P-value |
| --- | --- | --- | --- | --- | --- | --- |
| WARS2-AS1 | 1.384059 | | 1.017249 | 1.883136 | 0.038563 | |
| SUV39H2-DT | 1.604315 | | 1.19715 | 2.149962 | 0.001553 | |
| LINC00623 | 1.258436 | | 1.087219 | 1.456616 | 0.002065 | |
| SNHG7 | 1.032731 | | 1.007928 | 1.058145 | 0.009413 | |
| ZNF8-DT | 1.389317 | | 1.131248 | 1.706259 | 0.001712 | |
| HPN-AS1 | 0.752593 | | 0.584548 | 0.968948 | 0.027479 | |
| MIR4435-2HG | 1.12979 | | 1.049062 | 1.216731 | 0.001254 | |
| NRSN2-AS1 | 1.291849 | | 1.093971 | 1.52552 | 0.002538 | |
| CYTOR | 1.036757 | | 1.013248 | 1.060811 | 0.002038 | |
| BACE1-AS | 1.226284 | | 1.109387 | 1.3555 | 6.58E-05 | |
| MAPKAPK5-AS1 | 1.071114 | | 1.013056 | 1.1325 | 0.015685 | |
| STARD7-AS1 | 1.448668 | | 1.002626 | 2.093143 | 0.04839 | |
| ZNF433-AS1 | 1.935442 | | 1.188432 | 3.151997 | 0.00796 | |
| HNRNPD-DT | 2.407334 | | 1.268186 | 4.56972 | 0.00722 | |
| RUSC1-AS1 | 1.121147 | | 1.006607 | 1.248721 | 0.037551 | |
| SCAT2 | 1.402795 | | 1.117264 | 1.761297 | 0.003558 | |
| MYLK-AS1 | 1.275519 | | 1.047072 | 1.553809 | 0.015659 | |
| HEATR6-DT | 1.305749 | | 1.004622 | 1.697136 | 0.046104 | |
| PXN-AS1 | 1.191045 | | 1.046708 | 1.355286 | 0.007988 | |
| DNAJC9-AS1 | 2.705893 | | 1.654415 | 4.425648 | 7.32E-05 | |
| MELTF-AS1 | 1.163105 | | 1.045494 | 1.293947 | 0.005471 | |
| SBF2-AS1 | 1.466437 | | 1.168308 | 1.840643 | 0.000962 | |
| SNHG12 | 1.144727 | | 1.045235 | 1.253688 | 0.003572 | |
| SNHG4 | 1.485983 | | 1.279163 | 1.726243 | 2.22E-07 | |
| MYG1-AS1 | 1.475288 | | 1.16184 | 1.8733 | 0.001418 | |
| HCG15 | 2.325228 | | 1.357253 | 3.983552 | 0.002126 | |
| C2orf49-DT | 2.598096 | | 1.393904 | 4.84259 | 0.002653 | |
| ASH1L-AS1 | 1.262652 | | 1.010526 | 1.577683 | 0.04016 | |
| PRANCR | 1.300211 | | 1.009407 | 1.674794 | 0.042108 | |
| LINC01011 | 2.026211 | | 1.331488 | 3.083415 | 0.000979 | |
| ZBTB11-AS1 | 2.290395 | | 1.436038 | 3.653043 | 0.000503 | |
| FAM111A-DT | 1.592828 | | 1.159121 | 2.188816 | 0.004098 | |
| MED8-AS1 | 2.638414 | | 1.631964 | 4.265553 | 7.55E-05 | |
| LINC00944 | 1.212386 | | 1.038376 | 1.415558 | 0.014836 | |
| ZEB1-AS1 | 1.479356 | | 1.189269 | 1.840201 | 0.000437 | |
| NCK1-DT | 1.588408 | | 1.267124 | 1.991155 | 5.99E-05 | |
| LINC00653 | 1.798739 | | 1.013875 | 3.191184 | 0.044742 | |
| HMGN3-AS1 | 2.295671 | | 1.541002 | 3.419921 | 4.38E-05 | |
| ZNF232-AS1 | 1.076541 | | 1.003594 | 1.15479 | 0.039381 | |
| OTUD6B-AS1 | 1.114522 | | 1.014879 | 1.223948 | 0.023265 | |
| TFAP2A-AS1 | 1.486308 | | 1.089482 | 2.027671 | 0.012392 | |
| PSPC1-AS2 | 1.867298 | | 1.07687 | 3.237905 | 0.026171 | |
| ZNF32-AS2 | 1.856634 | | 1.111718 | 3.100688 | 0.018045 | |
| GAS5 | 1.005388 | | 1.000477 | 1.010323 | 0.031474 | |
| SNHG1 | 1.046342 | | 1.019147 | 1.074262 | 0.000747 | |
| SREBF2-AS1 | 1.500209 | | 1.199736 | 1.875935 | 0.000375 | |
| SNHG3 | 1.074262 | | 1.044166 | 1.105225 | 7.77E-07 | |
| CAPN10-DT | 1.753169 | | 1.089807 | 2.820317 | 0.02064 | |
| MAFG-DT | 1.072939 | | 1.027076 | 1.120851 | 0.001586 | |
| NRAV | 1.252756 | | 1.14606 | 1.369384 | 6.99E-07 | |
| ZNF529-AS1 | 1.321042 | | 1.05891 | 1.648065 | 0.013618 | |
| SUGT1-DT | 1.729627 | | 1.05779 | 2.82817 | 0.02897 | |
| ZNF32-AS1 | 1.886873 | | 1.081767 | 3.29118 | 0.025295 | |
| LINC00294 | 1.175644 | | 1.005958 | 1.373952 | 0.041885 | |
| TTC23L-AS1 | 1.54638 | | 1.066245 | 2.242721 | 0.021555 | |
| LINC00862 | 1.352992 | | 1.130545 | 1.619207 | 0.000971 | |
| PITPNA-AS1 | 1.064772 | | 1.006123 | 1.126841 | 0.029921 | |
| PTOV1-AS1 | 1.282672 | | 1.086888 | 1.513722 | 0.00322 | |
| NIFK-AS1 | 1.296657 | | 1.085229 | 1.549277 | 0.004229 | |
| MKLN1-AS | 3.487362 | | 2.328314 | 5.223391 | 1.36E-09 | |
| KPNB1-DT | 2.989059 | | 1.32575 | 6.739185 | 0.008296 | |
| SNHG20 | 1.282348 | | 1.065572 | 1.543224 | 0.008484 | |
| LINC00205 | 1.253889 | | 1.116063 | 1.408734 | 0.00014 | |
| LINC00665 | 1.134202 | | 1.052581 | 1.222152 | 0.00095 | |
| SNHG21 | 1.803518 | | 1.313282 | 2.476755 | 0.000269 | |
| SNHG14 | 1.300891 | | 1.094834 | 1.54573 | 0.002792 | |
| NAV2-AS6 | 1.228468 | | 1.021248 | 1.477733 | 0.029033 | |
| LINC00839 | 1.113534 | | 1.018136 | 1.217871 | 0.018609 | |
| LENG8-AS1 | 1.284934 | | 1.041938 | 1.584601 | 0.019074 | |
| GIHCG | 1.11276 | | 1.022114 | 1.211445 | 0.013721 | |
| DGUOK-AS1 | 1.508102 | | 1.065056 | 2.135447 | 0.020606 | |
