# Supplementary material for: Prognosis and Tumour Immune Microenvironment of Patients With Hepatocellular Carcinoma by a Novel Pyroptosis-Related lncRNA Signature
Source: Front Immunol. 2022 Jun 24;13:836576. doi: 10.3389/fimmu.2022.836576 (PMC9263208; doi:10.3389/fimmu.2022.836576)
Supplement: Supplementary file 4 [file Table_4.docx]

| id | coef | HR | HR.95L | HR.95H | P-value |
| --- | --- | --- | --- | --- | --- |
| HPN-AS1 | -0.49472260  5197514 | 0.609740022552502 | 0.413297985430753 | 0.899551675082184 | 0.0126484594971844 |
| MED8-AS1 | 0.828791005940868 | 2.29054780689002 | 1.18813654442082 | 4.41583021773499 | 0.0133350735971851 |
| ZNF232-AS1 | 0.0809636586791231 | 1.08433148981211 | 0.996013916023365 | 1.18048027330029 | 0.0617869211619114 |
| SREBF2-AS1 | 0.274236888700321 | 1.31552639866925 | 0.981409272044318 | 1.7633924550058 | 0.0665893432138089 |
| MKLN1-AS | 0.958543438224837 | 2.6078951452783 | 1.47058876037954 | 4.62475796905375 | 0.00104024225605537 |
